# The role of voluntary health insurance in frailty onset and progression among older and aging European adults: a survival analysis, 2013–2022

**DOI:** 10.3389/fpubh.2026.1880660

**Published:** 2026-09-01

**Authors:** Michele Sorrentino, Yamato Uejima, Giuseppina Affinito, Paolo Montuori, Raffaele Palladino

**Affiliations:** 1 Department of Public Health, University of Naples Federico II, Naples, Italy; 2 PhD National Program in One Health Approaches to Infectious Diseases and Life Science Research, Department of Public Health, Experimental and Forensic Medicine, University of Pavia, Pavia, Italy; 3 Department of Primary Care and Public Health, School of Public Health, Imperial College, London, United Kingdom; 4 Interdepartmental Research Center in Healthcare Management and Innovation in Healthcare (CIRMIS), Naples, Italy

**Keywords:** aging, Europe, frailty, SHARE, voluntary health insurance

## Abstract

**Background:**

Frailty is a syndrome characterized by physiological decline and increased vulnerability to adverse health outcomes, including disability, hospitalization, and mortality In Europe, 5.8–27.3% of those aged ≥65 is frail, and up to 50.9% is pre-frail. With an aging population, and cuts to public health spending, erosion of universal health coverage is becoming more evident in European Health Systems. As a result, voluntary health insurance (VHI) is becoming more common, offering faster access and reduced out-of-pocket expenses. However, VHI may also widen health inequalities, as lower-income groups face greater barriers to care, delayed or missed care, and prevention, increasing their risk of becoming frail.

**Aim:**

This study aimed to estimate the prevalence of VHI and assess its impact on the progression and onset of frailty among Europeans aged 50 and older between 2013 and 2022.

**Methods:**

Data was retrieved from waves 5, 6, 8, and 9 of Survey of Health, Aging and Retirement in Europe (SHARE) dataset, a longitudinal panel study of adults aged 50 years and older. Frailty was assessed using the SHARE Frailty Instrument (SHARE-FI), which includes weight loss, exhaustion, grip strength, walking speed, and physical activity. Mixed effects linear regression and Cox proportional hazards regression were employed to assess the impact of VHI on frailty. To further assess whether the association varied across subgroups, analyses were stratified by income quartiles (Q1 to Q4 being the highest) and country of residence.

**Results:**

At baseline 191,848 individuals aged 50 and above from 17 counties were included. VHI coverage was 37%, and increased across survey waves, with marked heterogeneity by country of residence and quartile of income. VHI was associated with lower frailty scores (coeff. = −0.056; 95% CI: −0.071 to −0.041) with the strongest association observed in Q1 (coeff.:= − 0.786; 95% CI: −0.817 to −0.755). Country-specific analyses confirmed this figure. When analyzing the risk of frailty onset (*n* = 53,873; 311,695 person-years) 4,645 events were recorded. VHI was associated with a reduced risk of frailty onset (HR = 0.862; 95% CI: 0.763 to 0.973), particularly in Q2 (HR = 0.798; 95% CI: 0.667 to 0.955) and in Spain (HR = 0.626; 95% CI: 0.475 to 0.824).

**Conclusion:**

VHI was associated with lower frailty progression and reduced risk of frailty onset. Nonetheless, being an out-of-pocket expense, it also might increase inequalities by weakening the Public Health System if not fully integrated into a wider strategy.

## Introduction

Frailty is a syndrome characterized by physiological decline and increased vulnerability to adverse health outcomes, including disability, hospitalization, and mortality (1–3). The prevalence of frailty has considerably increased globally over the last decade (4), with a disproportionate burden for lower income subgroups (5). In Europe, the prevalence of frailty among individuals aged 65 years and older ranges from 5.8 to 27.3%, while up to 50.9% are classified as pre-frail (6).

The rising proportion of older people (7), and cuts in public spending for healthcare have raised concern over the sustainability and equity of universal health coverage. For this reason, voluntary health insurance (VHI) is becoming progressively more common by offering faster access to care and mitigating out-of-pocket expenses (8). VHI refers to a discretionary form of coverage that individuals can purchase to supplement or complement publicly financed health systems (9). Unlike the US, European healthcare systems are generally publicly funded; however, the proportion differs significantly within the countries (10). In this context, VHI may exacerbate existing health inequalities, as lower-income individuals are more likely to encounter delays, barriers to preventive services, and unmet healthcare needs, factors that could increase their vulnerability to frailty (11). Nevertheless, by improving access to timely diagnostics, chronic disease management, and preventive services, VHI might mitigate frailty risk by addressing key physiological, behavioral, and social determinants of decline.

Despite increasing reliance on VHI across European countries, its implications for vulnerable aging populations, particularly regarding health equity, remain poorly understood and underexplored in comparative research. While previous studies have examined the association between VHI and health outcomes such as mortality or multi-morbidity (12, 13), no study to date has specifically investigated the role of VHI in the onset or progression of frailty in the European population. Nonetheless, frailty onset and progression allow to better assess the impact on health in daily life, serving as a valuable proxy for overall quality of life. Leveraging longitudinal data from a large, representative European cohort, this study aims to estimate the prevalence of VHI in Europe, and assess whether VHI influences the progression and onset of frailty among adults aged 50 years and older since 2013. Understanding the role of VHI in shaping frailty trajectories is essential for informing equitable aging policies and anticipating potential unintended consequences of health system privatization.

## Methods

### Study population

This study utilized data from waves 5 (2013), 6 (2015), 8 (2019/2020), and 9 (2021/2022) of the Survey of Health, Aging and Retirement in Europe (SHARE), a longitudinal panel study of individuals aged 50 and older conducted across 28 European countries and Israel (13–17). These waves were selected to ensure consistency in the measurement of voluntary health insurance (VHI), as relevant survey items underwent substantial revisions between waves 1 and 5. SHARE provides data on health status, household income, VHI coverage, and sociodemographic indicators.

However, several methodological constraints influenced data availability across waves. Wave 7, was excluded because approximately 80% of participants completed the SHARELIFE retrospective life-history interview, which omitted VHI and other essential variables (18). In addition, some countries did not collect standard SHARE data in specific waves due to administrative or logistical changes; for example, the shift to online survey administration in the Netherlands during wave 6 and 7 (18). For this reason, the final analytical sample included 17 countries that participated in at least one of the selected waves and demonstrated sufficient data completeness and consistency.

Missing data were handled using a deterministic imputation strategy when information was available from others SHARE waves or from equivalent variables collected in other sections of the questionnaire. This approach was applied only to selected covariates with limited missingness. Participants with missing values for frailty status, or other key covariates after this procedure were excluded from the final analytical sample. The exact number and proportion of missing data is fully provided for transparency in the Supplementary material S1. Given the observational nature of the study, residual bias due to missing data cannot be excluded.

### Voluntary health insurance

The exposure variable was the status of VHI. Participants were asked to respond “yes” or “no” to the following question: “Do you have any supplementary health insurance that pays for services not covered by your basic health insurance/national health system/third-party payer?” This item captures whether respondents hold additional insurance coverage beyond their primary public or statutory health coverage (e.g., social health insurance or national health systems).

### Outcome

The primary outcome of interest was frailty. Participants frailty was assessed using the SHARE Frailty Instrument (SHARE-FI), derived from the Fried frailty phenotype and specifically developed for use within the SHARE study (19). The SHARE-FI is a significant predictor of incident disability and mortality, with performance comparable to more complex frailty indices requiring clinical assessment (20). SHARE-FI incorporates five domains: exhaustion, weight loss, grip strength, walking difficulties, and physical activity. Following the methodology proposed by Romero-Ortuno et al., a continuous frailty score (DFS) was calculated and used to evaluate frailty progression over time (21). Lower values indicate lower levels of frailty, whereas higher values indicate greater frailty. In addition, participants were categorized as non-frail, pre-frail, or frail using the sex-specific SHARE-FI thresholds. In particular, for female if predicted DFS < 0.31, (non-frail), if predicted DFS < 2.1301121973, (pre-frail), if predicted DFS < 6, (frail), for male if predicted DFS < 1.211878526, (non-frail), if predicted DFS < 3.0052612772, (pre-frail), if predicted DFS < 7, (frail) to assess change of status of the participant. The continuous score was used in mixed-effects models evaluating frailty progression, whereas the categorical classification was used in survival analyses assessing incident frailty.

### Covariates

Demographic covariates included age, gender (male/female), and marital status (categorized as married/in a registered partnership or other). Socioeconomic variables comprised total household income pro capita (divided into country- and wave-specific quartiles, with Q1 and Q4 representing the lowest and highest income groups, respectively), employment status (employed/self-employed, retired, or other), and educational attainment (none primary/lower secondary, upper secondary, or post-secondary/tertiary). Health behaviors included present or past smoking status (ever smoked: yes/no) and frequency of vigorous physical activity (categorized as hardly ever/never or once a month or more). Due to a routing error in wave 6, approximately 40% of participants had missing smoking data; this was addressed in sensitivity analyses through inclusion of a missingness indicator. Health status variables included body mass index (BMI), calculated from self-reported height and weight, and the number of self-reported chronic diseases. Country of residence was also included as a covariate. The year in which the interview was taken was used to estimate the year of follow up.

### Statistical analysis

Descriptive statistics were employed to describe the baseline population, as appropriate. Weighted prevalence of frailty and VHI was calculated using the calibrated cross-sectional weights provided by the SHARE. Descriptive statistics were employed, as appropriate.

We conducted a mixed-effects linear regression model to investigate predictors of disability-free survival (DFS; continuous outcome). A random intercept at the patient level was incorporated to account for within-subject correlation due to repeated measures and to address potential heteroscedasticity. Country and interview year were included as fixed effects to adjust for contextual and temporal variation. Other independent variables in the model included age, gender, marital status, income quartile pro capita, job, education, smoking habits, vigorous physical activity, BMI, number of diseases, country of residence. To further assess whether the association varied across income groups, analyses were stratified by income quartiles. Separate analyses were also conducted for each country included, considering the differences within European Health Systems. Also, to further assess the potential heterogeneity related to health inequalities within countries, a sensitive analysis was performed controlling for GINI index. When available, the index was paired to each record for country and year of interview; data without a year-specific value were dismissed. Model diagnostics included log-likelihood tests, Wald statistics, and estimation of variance components for random effects and residual error. All analyses were conducted on complete cases.

A secondary analysis employed a Cox proportional hazards regression model to examine the association between VHI and the risk of frailty onset, correcting the analyses for multiple sociodemographic, behavioral, and health-related factors. The outcome variable was time to event (failure), defined as the first transition from non-frail to frail status during the follow-up period. Survival time was constructed using longitudinal SHARE data, which allows linkage of respondents across waves. Independent variables included VHI status as well as all the other covariates included in the first model. The Cox model was estimated using the Breslow method to handle tied events (22). The proportional hazards assumption was assessed using Schoenfeld residual and log–log plots. Potential multicollinearity was assessed using the correlation matrix of coefficients. In line with the previous outcome, analyses were also conducted for income quartiles and countries separately, and a sensitive analysis controlling for GINI index. Hazard ratios (HRs), 95% confidence intervals (CIs), and *p*-values were reported for each covariate. Stata software, version 18.0 was used for all analyses.

### Ethics

Ethical approval for the SHARE study was granted by the Ethics Council of the Max Planck Society. This analysis was conducted using de-identified, publicly available data and complies with institutional guidelines for secondary use of anonymized datasets.

## Results

### Baseline characteristics

**Figure 1:**
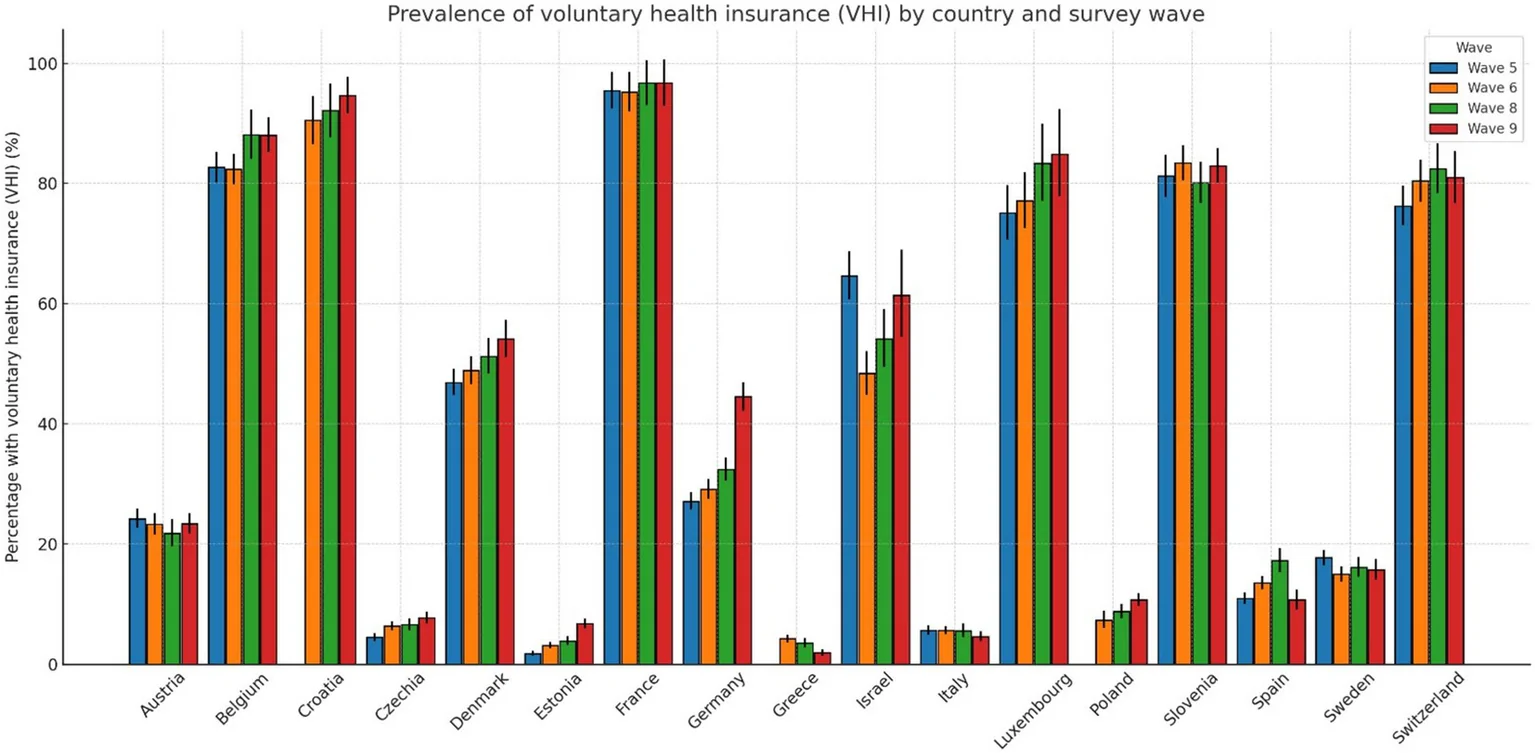
Prevalence of voluntary health insurance among baseline population. Prevalence of voluntary health insurance among individuals aged 50 and older by country of residence across wave of participation (wave 5 to 9, excluding 7). Each bar represents the percentage of individuals with voluntary health insurance. This data refers to cross-sectional weighted database. For some countries data was unavailable for specific waves.

The study population included 191,848 individuals from 17 countries between 2013 and 2022: 52,119 from Wave 5, 56,770 from Wave 6, 36,583 from Wave 8, and 46,376 from Wave 9. The overall mean age was 67.91 years, ranging from 65.9 in Luxembourg to 70.5 in Sweden. Women accounted for 55.26% of the sample, with their proportion increasing from 54.34% in Wave 5 to 56.16% in Wave 9. Overall, 76,680 participants (39.97%) had VHI, a share that grew steadily from 38.18% in Wave 5 to 43.16% in Wave 9. Country-level variation was notable: France (95.2%), Croatia (92.94%), and Belgium (84.62%) had the highest insurance prevalence, while Italy (5.39%), Estonia (3.74%), and Greece (3.41%) had the lowest. Country-level differences in voluntary insurance prevalence are displayed in Figure 1. Voluntary health insurance coverage was more common in higher-income groups, reaching 45.42% in the top quartile and 34.65% in the bottom, a pattern consistent across most countries.

Table 1 presents characteristics by insurance status using weighted data. Of the sample, 37.1% subscribed a voluntary health insurance while 62.9% did not. At baseline, insured individuals were slightly younger (65.4 vs. 66.1 years) and less likely to be frail (5.8% vs. 8.7%). Gender distribution was identical (47.7% male, 52.3% female). The insured more often reported being married or in a registered partnership (66.7% vs. 65.4%), had higher income (30.6% in Q4 vs. 24.6%), and greater educational attainment (35.1% tertiary education vs. 22.3%). Only 24.2% of insured individuals had less than upper secondary education, compared to 41.1% among the uninsured.

**Table 1:** Baseline characteristics by voluntary health insurance using weights across 17 countries.

	No VHI	With VHI	Total
*N*	62.9%	37.1%	191,848 (100.0%)
Frailty status at baseline			
Non-Frail	73.7%	80.5%	76.2%
Pre-Frail	17.6%	13.7%	16.2%
Frail	8.7%	5.8%	7.6%
Age at interview (in years)	66.1 (10.2)	65.4 (10.2)	65.8 (10.2)
Gender			
Male	47.7%	47.7%	47.7%
Female	52.3%	52.3%	52.3%
Marital status			
Other	34.6%	33.3%	34.1%
Married or in a registered partnership	65.4%	66.7%	65.9%
Household income			
Q1	27.8%	21.6%	25.5%
Q2	24.4%	22.5%	23.7%
Q3	23.1%	25.3%	23.9%
Q4	24.6%	30.6%	26.8%
Current job situation			
Employed or self-employed	31.6%	38.0%	33.9%
Retired	48.0%	51.5%	49.3%
Other	20.4%	10.5%	16.8%
Education			
Less than upper secondary	41.1%	24.2%	34.9%
Upper secondary	36.5%	40.7%	38.0%
Above	22.3%	35.1%	27.1%
Smoking habits (present or past)			
No	53.9%	52.1%	53.2%
Yes	46.1%	47.9%	46.8%
Vigorous physical activity			
Hardly ever, or never	44.5%	40.1%	42.9%
Once a month or more	55.5%	59.9%	57.1%
Body mass index (kg/m^2^)			
<18.5	1.1%	1.6%	1.3%
18.5–24.9	35.4%	39.7%	37.0%
25–29.9	41.8%	39.5%	40.9%
30+	21.7%	19.2%	20.8%
Number of chronic diseases	1.8 (1.7)	1.7 (1.6)	1.8 (1.7)
Number of medications	1.6 (1.6)	1.4 (1.5)	1.5 (1.6)

Data are expressed as arithmetic mean (standard deviation) for continuous variables. Data across wave were merged for this analysis. Cross-sectional weights were applied. Regarding household income Q1 represent the lowest, and Q4 represent the highest income quartile. VHI, voluntary health insurance.

Daily smoking was similar across groups (~47%), but insured individuals were more likely to engage in regular vigorous activity (59.9% vs. 55.5%). A higher share of insured individuals had normal BMI (39.7% vs. 35.4%), and a lower share were obese (19.2% vs. 21.7%). They also reported fewer chronic diseases (1.7 vs. 1.8) and took fewer medications (1.4 vs. 1.6).

### Association of voluntary health insurance with frailty

The presence of voluntary health insurance (VHI) was significantly associated with lower levels of frailty (DFS score). Overall, individuals with VHI had a mean DFS score that was 0.06 points lower than those without insurance (coeff. = − 0.056, 95% CI: −0.071 to −0.041). Stratified analyses by income quartiles revealed that the association was strongest among individuals in the lowest income group (Q1), with a substantially larger effect (coeff. = − 0.079, 95% CI: −0.0111 to −0.047), while significant but smaller associations were observed in Q2 (coeff. = − 0.046, 95% CI: −0.078 to −0.015), Q3 (coeff. = − 0.040, 95% CI: −0.068 to −0.011), and Q4 (coeff. = − 0.063, 95% CI: −0.087 to −0.038).

Country-specific models indicated heterogeneity in the relationship between VHI and frailty. Significant negative associations indicating better health among the insured—were observed in Belgium (coeff. = − 0.140, 95% CI: −0.189 to −0.091), Germany (coeff. = − 0.078, 95% CI: −0.112 to −0.043), Denmark (coeff. = − 0.040, 95% CI: −0.077 to −0.004), France (coeff. = − 0.107, 95% CI: −0.205 to −0.009), Israel (coeff. = − 0.105, 95% CI: −0.175 to −0.035), Luxembourg (coeff. = − 0.161, 95% CI: −0.238 to −0.084), Slovenia (coeff. = − 0.124, 95% CI: −0.172 to −0.076), Spain (coeff. = − 0.105, 95% CI: −0.167 to −0.044), and Switzerland (coeff. = − 0.143, 95% CI: −0.189 to −0.096). Notably, Poland showed a significant positive association (coeff. = 0.148, 95% CI: 0.065 to 0.230), suggesting higher frailty among those with VHI. No significant associations were detected the other countries. These figure were confirmed when controlling for GINI index, a complete description of the results is available in Supplementary materials.

**Figure 2:**
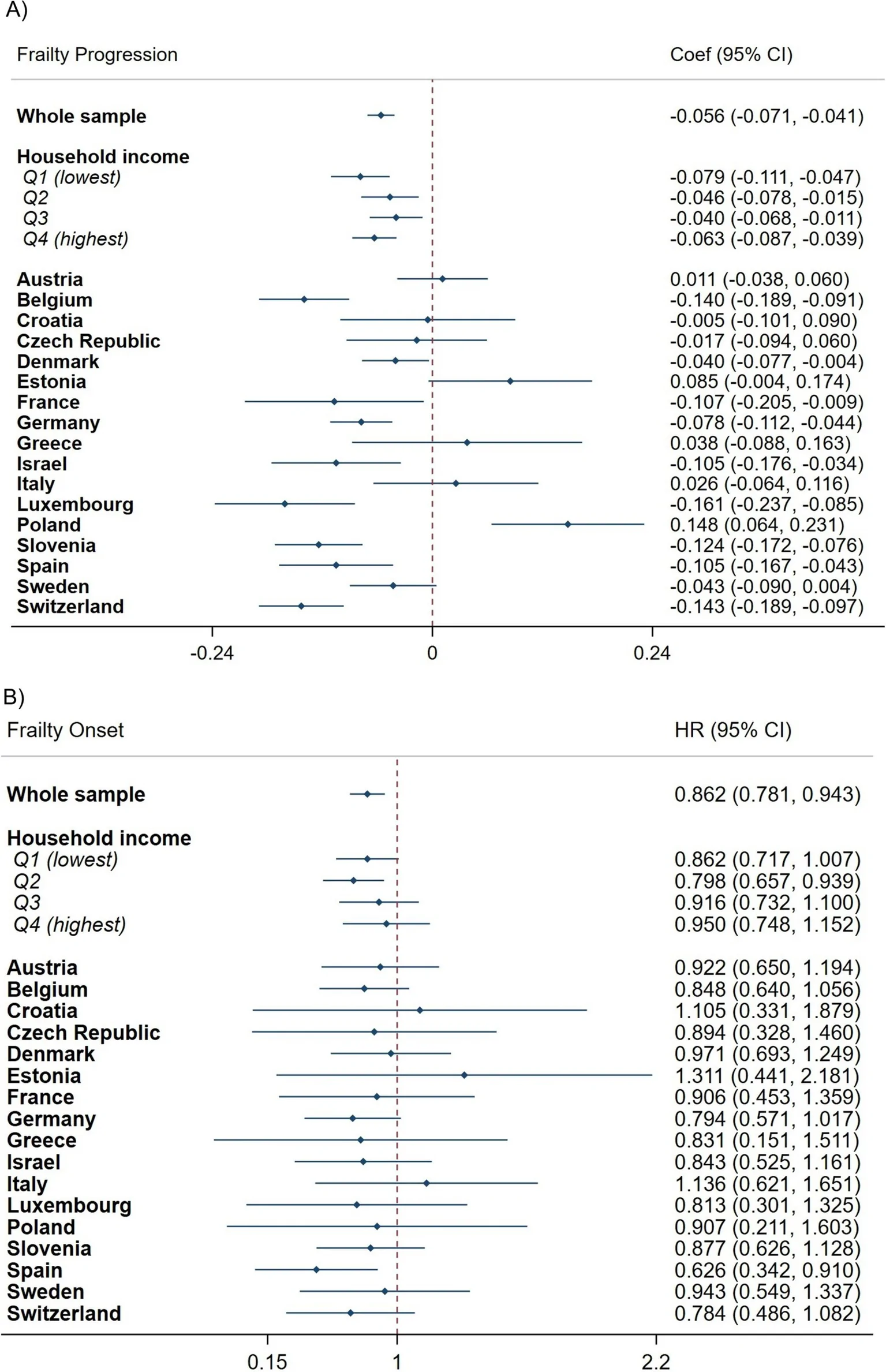
Coefficient, and hazard ratio for frailty progression **(A)** and onset **(B)** associated with voluntary health insurance. This figure presents coefficient, and hazard ratio (HR) with a 95% confidence interval (CI) using mixed effect linear regression and cox regression models. A stratified analysis was performed by quartiles of income and country of residence. The figure displays only the effect of the main variable (VHI) on the outcome (frailty progression, or DFS, and risk of frailty onset). The outcome in **(A)** is frailty progression, defined as increase in DFS, while in **(B)** is frailty onset, defined as a change of state from a condition of non-frailty to frailty. This model is adjusted for age, gender, marital status, household income, employment status, education, vigorous physical activities, body mass index, number of chronic diseases, and country of residence.

When analyzing the impact of VHI on frailty on-set, 53,873 individuals were included. The follow-up period ranged from 0 to 9 years, yielding a cumulative observation time of 311,695 person-years, during which 4,645 incident frailty cases were recorded. The mean age at baseline was 64.87 years, with 54.19% of participants being female. A total of 27.10% belonged to the highest socioeconomic status group. The proportional hazards assumption was assessed using Schoenfeld residual tests and graphical inspection of log–log survival plots. No substantial violations of the proportional hazards assumption were detected for the primary exposure variable (Supplementary material S1). In the overall sample, having VHI was associated with 14% lower risk of developing frailty (HR = 0.862, 95% CI: 0.763 to 0.973, *p* = 0.004). When stratifying by income quartiles, a protective association was most evident in the second quartile (Q2), where individuals with VHI had a 20% reduced risk of developing frailty compared to uninsured peers (HR = 0.798, 95% CI: 0.667 to 0.955, *p* = 0.013). No association was found in the other quartiles. A protective effect was observed only in Spain, where individuals with VHI had a 37% lower hazard of developing frailty (HR = 0.626, 95% CI: 0.475 to 0.824, *p* = 0.044). Results of these analysis are displayed in Figure 2. In the sensitive analysis, when controlling also for GINI index, these figures were confirmed (Supplementary material).

## Discussion

Among 191,848 older adults from 16 European countries and Israel (2013–2022), 37% had voluntary health insurance, with wide variation across countries, above 95% in France and little under 4% in Estonia and Greece, and higher prevalence in higher quartiles of income, 45% in the highest quartile (Q4) vs. 34% in the lowest. Frailty affected 7.6% of participants and was more common among the uninsured (8.7% vs. 5.8%). Overall, VHI was associated with a modest protective effect on frailty progression, confirmed when stratifying for each quartile of income and for the vast majority of the countries. VHI was also associated with a 14% reduction in risk of frailty onset. For those in the second income quartile and those living in Spain the association was even stronger (20 and 37%, respectively). No association was found in other sub-group analyses.

These results about the association between VHI and frailty progression are in line with previous evidence, although conducted in a different setting (23). This figure underlines the role of VHI, not only to prevent the occurrence of hard outcomes as previously evidenced (24), but also to prevent worse quality of life. Similarly, findings about the risk of frailty onset by VHI status confirm what has been previously found, although in a different setting (25).

The association between VHI and lower frailty progression as well as reduced risk of frailty onset may reflect several mechanisms, including improved access to healthcare services, preventive care, and chronic disease management. However, these findings should be interpreted cautiously because individuals purchasing VHI may differ systematically from uninsured individuals in ways that are not fully captured by the available covariates. Residual confounding related to health-seeking behaviors, health literacy, preventive care utilization, and other socioeconomic characteristics may partly explain the observed associations.

VHI might facilitate access to preventive services, specialized care, and reduce the burden of out-of-pocket expenses or delays in care, offering clear advantages over being uninsured. However, it may also contribute to widening health inequalities. The protective effect of VHI was more pronounced among individuals in the lowest income quartile, underscoring that the cost of subscribing to additional insurance may exacerbate existing inequalities. These dynamic risks deepening entrenched inequalities in health outcomes across income groups. Therefore, while VHI appears to mitigate frailty risk, close monitoring and appropriate regulation are necessary to minimize its potential to reinforce social and economic health inequities. Several caveats merit discussion. An important limitation is the potential for healthy-user and selection bias. Individuals who purchase voluntary health insurance may be more proactive in managing their health, more engaged with preventive care services, or possess higher levels of health literacy than uninsured individuals. Although our analyses adjusted for a wide range of sociodemographic, behavioral, and health-related factors, residual confounding from unmeasured characteristics cannot be excluded. Reverse causation should also be considered. Although VHI status was assessed before subsequent frailty measurements within the longitudinal framework, healthier individuals may be more likely to purchase and maintain voluntary insurance coverage. Consequently, the temporal relationship observed in this study does not establish a causal effect of VHI on frailty trajectories.

We also acknowledge that deterministic imputation is less robust than multiple imputation approaches and may introduce bias if data are not missing completely at random.

Then, the follow-up period for the longitudinal analyses may be insufficient to fully capture the long-term effects of VHI on frailty and disability-free survival, particularly given the progressive nature of age-related decline. Also, reliance on self-reported data may introduce information bias; for example, under-reporting of chronic conditions may be more common among older adults from disadvantaged backgrounds. Furthermore, recall-bias, and misreporting have to be considered being SHARE a survey-based dataset. Finally, being held across Europe response rates varied by countries, with some areas being more affected than others. However, the SHARE project employs robust methodology in both data collection and reporting, and for the present study, data selection, curation, and management were carefully conducted to reduce this bias. Third, the sample size in some sub-analyses was relatively small, which may have influenced certain results. Nonetheless, the SHARE database remains one of the largest datasets available in Europe, and these findings contribute valuable evidence for monitoring the impact of complementary financial strategies to improve access to care. Future research may benefit from further analyses focused on specific contexts, such as individual countries. Finally, the measurement of voluntary insurance did not account for variations in coverage type, benefits, or out-of-pocket costs, potentially leading to exposure misclassification and underestimation of heterogeneity in its protective effects. Also, the impact of voluntary health insurance may vary based on the inequalities within each country, while the use of an index might have reduced this limitation, it was beyond the current aim of this research to assess national differences in health policies. Nonetheless, to partially account for this limitation, all main analyses were conducted using weighted data to ensure representativeness, and results were stratified by income quartile and country to explore contextual differences in the effect of voluntary health insurance.

## Conclusion

This study identified an association between voluntary health insurance coverage and lower frailty progression as well as reduced risk of incident frailty among older adults in Europe. However, the observational design precludes causal inference, and the findings may partly reflect residual confounding, selection bias, and differences in healthcare-seeking behavior between insured and uninsured individuals.

While the findings of this study support the potential of voluntary insurance coverage as a complementary tool to promote healthy aging and reduce frailty, it also might widen the inequality for those in different socio-economic groups. Further research is needed to explore the long-term effects and to account for the variability in insurance coverage across contexts, a more equitable access to essential care services and prevention programs might help to tackle potential health inequalities. Moreover, the impact of COVID pandemic, which disproportionally affected older individual, should be further analyzed in terms of impact of voluntary health insurance to assess his role on frailty. Finally, future studies need to take in account country specific indexes of health inequalities to better understand the role of voluntary health insurance in reducing frailty burden.

## Data Availability

The datasets presented in this study can be found in: SHARE project, www.share-project.org. SHARE provides data under request after assessment.
