# Supplementary material for: The role of voluntary health insurance in frailty onset and progression among older and aging European adults: a survival analysis, 2013–2022
Source: Front Public Health. 2026 Sep 1;14:1880660. doi: 10.3389/fpubh.2026.1880660 (PMC13574600; doi:10.3389/fpubh.2026.1880660)
Supplement: Supplementary file 1 [file Table_1.DOCX]

| ***insurance*** | **DFS** | **STD ERROR** | **Z** | **p-value** | **[95% conf. interval]** | |
| --- | --- | --- | --- | --- | --- | --- |
| **Yes** | -0,547 | 0,008 | -7,160 | 0,000 | -0,070 | -0,040 |
| **age** | 0,039 | 0,000 | 94,660 | 0,000 | 0,038 | 0,040 |
|  |  |  |  |  |  |  |
| ***gender*** |  |  |  |  |  |  |
| **female** | -0,217 | 0,007 | -32,800 | 0,000 | -0,230 | -0,204 |
|  |  |  |  |  |  |  |
| ***marital*** |  |  |  |  |  |  |
| **Married or in a registered partnership** | 0,130 | 0,007 | -19,170 | 0,000 | -0,143 | -0,117 |
|  |  |  |  |  |  |  |
| ***income_pc_q*** |  |  |  |  |  |  |
| **Q2** | -0,054 | 0,007 | -7,710 | 0,000 | -0,067 | -0,040 |
| **Q3** | -0,101 | 0,007 | -14,060 | 0,000 | -0,115 | -0,087 |
| **Q4** | -0,133 | 0,007 | -17,780 | 0,000 | -0,148 | -0,119 |
|  |  |  |  |  |  |  |
| ***job*** |  |  |  |  |  |  |
| **Retired** | -0,175 | 0,008 | -21,640 | 0,000 | -0,191 | -0,159 |
| **Other** | 0,108 | 0,010 | 11,060 | 0,000 | 0,089 | 0,127 |
|  |  |  |  |  |  |  |
| ***education*** |  |  |  |  |  |  |
| **Upper secondary** | -0,159 | 0,008 | -19,700 | 0,000 | -0,175 | -0,144 |
| **Above** | -0,223 | 0,009 | -25,210 | 0,000 | -0,240 | -0,205 |
|  |  |  |  |  |  |  |
| ***eversmoked*** |  |  |  |  |  |  |
| **Yes** | 0,026 | 0,006 | 4,350 | 0,000 | 0,014 | 0,037 |
|  |  |  |  |  |  |  |
| ***sports*** |  |  |  |  |  |  |
| **Once a month or more** | -0,572 | 0,005 | -106,840 | 0,000 | -0,582 | -0,561 |
| **bmi2** | -0,035 | 0,004 | -9,150 | 0,000 | -0,042 | -0,027 |
| **disease** | 0,251 | 0,002 | 145,220 | 0,000 | 0,248 | 0,254 |
|  |  |  |  |  |  |  |
| ***country_mod*** |  |  |  |  |  |  |
| **Belgium** | 0,185 | 0,020 | 9,270 | 0,000 | 0,146 | 0,224 |
| **Croatia** | 0,236 | 0,020 | 11,970 | 0,000 | 0,197 | 0,274 |
| **Czechia** | 0,001 | 0,022 | 0,040 | 0,966 | -0,041 | 0,043 |
| **Denmark** | -0,026 | 0,020 | -1,340 | 0,180 | -0,065 | 0,012 |
| **Estonia** | 0,172 | 0,018 | 9,520 | 0,000 | 0,137 | 0,208 |
| **France** | 0,121 | 0,019 | 6,410 | 0,000 | 0,084 | 0,158 |
| **Germany** | -0,042 | 0,018 | -2,390 | 0,017 | -0,076 | -0,008 |
| **Greece** | 0,312 | 0,022 | 14,010 | 0,000 | 0,268 | 0,356 |
| **Israel** | 0,308 | 0,034 | 9,100 | 0,000 | 0,242 | 0,375 |
| **Italy** | 0,280 | 0,021 | 13,060 | 0,000 | 0,238 | 0,323 |
| **Luxembourg** | -0,077 | 0,026 | -3,000 | 0,003 | -0,127 | -0,027 |
| **Poland** | 0,154 | 0,019 | 8,060 | 0,000 | 0,116 | 0,191 |
| **Slovenia** | 0,188 | 0,024 | 7,840 | 0,000 | 0,141 | 0,235 |
| **Spain** | 0,133 | 0,023 | 5,880 | 0,000 | 0,089 | 0,177 |
| **Sweden** | -0,126 | 0,019 | -6,790 | 0,000 | -0,163 | -0,090 |
| **Switzerland** | -0,061 | 0,021 | -2,860 | 0,004 | -0,102 | -0,019 |
|  |  |  |  |  |  |  |
| **years** | -0,001 | 0,001 | -1,300 | 0,195 | -0,002 | 0,001 |
| **Gini** | 1580432,000 | 0,282 | 5,600 | 0,000 | 1027189,000 | 2133676,000 |

| ***insurance*** |  |  |  |  |
| --- | --- | --- | --- | --- |
| **yes** | 0,8717444 | 0,0413905 | -2,89 | 0,004 |
| **age** | 1,079867 | 0,002286 | 36,3 | 0 |
|  |  |  |  |  |
| ***gender*** |  |  |  |  |
| **female** | 1,32 | 0,046 | 8.03 | 0 |
|  |  |  |  |  |
| ***marital*** |  |  |  |  |
| **registered partenership** | 0,843 | 0,027 | -5,23 | 0 |
|  |  |  |  |  |
| ***income*** |  |  |  |  |
| **Q2** | 0,881 | 0,034 | -3,26 | 0,001 |
| **Q3** | 0,802 | 0,3325 | -5,32 | 0 |
| **Q4** | 0,719 | 0,33566 | -7,08 | 0 |
|  |  |  |  |  |
| ***job*** |  |  |  |  |
| **retired** | 1,35 | 0,082 | 4,95 | 0 |
| **other** | 1,91 | 0,125 | 9,8 | 0 |
|  |  |  |  |  |
| ***education*** |  |  |  |  |
| **upper secondary** | 0,854 | 0,031 | -4,28 | 0 |
| **above** | 0,667 | 0,0299 | -9,04 | 0 |
|  |  |  |  |  |
| ***eversmoked*** |  |  |  |  |
| **yes** | 1,252 | 0,03996 | 7,04 | 0 |
|  |  |  |  |  |
| ***sports*** |  |  |  |  |
| **once a month or more** | 0,555 | 0,0173 | -18,88 | 0 |
|  |  |  |  |  |
| ***bmi*** |  |  |  |  |
| **below 18,5** | 1,375 | 0,201 | 2,18 | 0,029 |
| **25-29.9** | 1,089 | 0,041 | 2,3 | 0,021 |
| **30 and above** | 1,614 | 0,0649 | 11,91 | 0 |
|  |  |  |  |  |
| **disease** | 1,249 | 0,01 | 26,89 | 0 |
|  |  |  |  |  |
| ***country*** |  |  |  |  |
| **Belgium** | 0,657 | 0,0823 | -3,36 | 0,001 |
| **Croatia** | 1,774 | 0,191 | 5,32 | 0 |
| **Czechia** | 0,396 | 0,0615 | -5,96 | 0 |
| **Denmark** | 0,5697 | 0,0646 | -4,96 | 0 |
| **Estonia** | 2,3 | 0,3321 | 5,77 | 0 |
| **France** | 1,687 | 0,175 | 5,14 | 0 |
| **Germany** | 1,14 | 0,0965 | 1,52 | 0,128 |
| **Greece** | 2,813 | 0,5289 | 5,5 | 0 |
| **Israel** | 9,521 | 2,869 | 7,48 | 0 |
| **Italy** | 2,859 | 0,4354 | 6,9 | 0 |
| **Luxembourg** | 0,8487 | 0,1308 | -1,06 | 0,287 |
| **Poland** | 1,067 | 0,1156 | 0,6 | 0,552 |
| **Slovenia** | 0,58 | 0,1 | -3,15 | 0,002 |
| **Spain** | 3,347 | 0,631 | 6,41 | 0 |
| **Sweden** | 0,4725 | 0,051 | -6,93 | 0 |
| **Switzerland** | 1,083 | 0,1301 | 0,66 | 0,609 |
|  |  |  |  |  |
| **Gini index** | 5,09 e-08 | 1,59 e07 | 5,39 | 0 |
